# Using Intervention Mapping for Program Design and Production of *iCHAMPSS*: An Online Decision Support System to Increase Adoption, Implementation, and Maintenance of Evidence-Based Sexual Health Programs

**DOI:** 10.3389/fpubh.2017.00203

**Published:** 2017-08-11

**Authors:** Melissa F. Peskin, Belinda F. Hernandez, Efrat K. Gabay, Paula Cuccaro, Dennis H. Li, Eric Ratliff, Kelly Reed-Hirsch, Yanneth Rivera, Kimberly Johnson-Baker, Susan Tortolero Emery, Ross Shegog

**Affiliations:** ^1^University of Texas Health Science at Houston School of Public Health, Houston, TX, United States; ^2^Northwestern University, Chicago, IL, United States; ^3^Harris County Public Health, Houston, TX, United States; ^4^University of Texas MD Anderson Cancer Center, Houston, TX, United States

**Keywords:** dissemination, evidence-based, intervention mapping, sexual health, adolescents

## Abstract

In Texas and across the United States, unintended pregnancy, HIV, and sexually transmitted infections (STIs) among adolescents remain serious public health issues. Sexual risk-taking behaviors, including early sexual initiation, contribute to these public health problems. Over 35 sexual health evidence-based programs (EBPs) have been shown to reduce sexual risk behaviors and/or prevent teen pregnancies or STIs. Because more than half of these EBPs are designed for schools, they could reach and impact a considerable number of adolescents if implemented in these settings. Most schools across the U.S. and in Texas, however, do not implement these programs. U.S. school districts face many barriers to the successful dissemination (i.e., adoption, implementation, and maintenance) of sexual health EBPs, including lack of knowledge about EBPs and where to find them, perceived lack of support from school administrators and parents, lack of guidance regarding the adoption process, competing priorities, and lack of specialized training on sexual health. Therefore, this paper describes how we used intervention mapping (Steps 3 and 4, in particular), a systematic design framework that uses theory, empirical evidence, and input from the community to develop *CH*oosing *A*nd *M*aintaining Effective *P*rograms for *S*ex Education in *S*chools (*iCHAMPSS*), an online decision support system to help school districts adopt, implement, and maintain sexual health EBPs. Guided by this systematic intervention design approach, *iCHAMPSS* has the potential to increase dissemination of sexual health EBPs in school settings.

## Introduction

In Texas and across the United States, unintended pregnancy, HIV, and sexually transmitted infections (STIs) among adolescents remain serious public health issues. In the U.S., the 2015 teen birth rate (among 15- to 19-year-old females) was 22.3 births per 1,000 (1). Furthermore, national estimates indicate that half of all new STIs occur among young people between the ages of 15 and 24 (2). Texas has one of the highest teen birth rates in the nation at 34.6 per 1,000 (3) and currently ranks sixth in the nation for the estimated number of HIV diagnoses among adults and adolescents (4). Sexual risk-taking behaviors, including early sexual initiation (5), are factors that contribute to these high rates of teen births and HIV/STIs (6–12).

National agencies, including the U.S. Department of Health and Human Services’ Office of Adolescent Health (13) and National Campaign to Prevent Teen and Unplanned Pregnancy (14), have developed online repositories (or lists) of evidence-based HIV, STI, and teen pregnancy prevention programs [hereafter referred to as sexual health evidence-based programs (EBPs)]. These programs are designated as *evidence-based* because they have been rigorously evaluated (usually in an experimental or quasi-experimental design) and shown to reduce sexual risk behaviors (e.g., sexual initiation, contraceptive use, frequency of sexual activity, and number of sexual partners) and/or prevent teen pregnancies or STIs (13). Examples of sexual health EBP categories from these online repositories include abstinence education, comprehensive sexual health education (where abstinence is stressed but information on contraception is also included), and positive youth development programs. Over 35 sexual health EBPs are designed for multiple settings (e.g., school, after school, clinic, and home), but more than half are school based (13).

The broad dissemination of sexual health EBPs in school settings could reach a considerable number of adolescents and help adolescents delay sexual initiation and reduce other sexual behaviors that increase their risk for unintended pregnancy, HIV, and STIs (15, 16). *Dissemination* is often used to describe the adoption, implementation, and maintenance process for the delivery of a new innovation. A sexual health EBP is an “innovation” because it may be “perceived as new by an individual or unit of adoption” (17). *Adoption* refers to the decision to use a particular innovation (17, 18). *Implementation* refers to the process of program use, often measured in terms of general use, completeness (how much of the program is taught), and fidelity (adherence to core program elements) (19, 20). *Maintenance*, or institutionalization, refers to the process whereby a program is integrated fully into the practices and activities of an organization (21, 22).

Most school districts across the U.S. and in Texas do not adopt and implement sexual health EBPs (15, 23, 24). In Texas, there are many individual- and school district-level barriers that impede dissemination of these programs (and non-EBPs), including lack of knowledge about EBPs and where to find them, perceived lack of support from school administrators and parents, lack of guidance regarding the adoption process, competing priorities, lack of specialized training on sexual health, misinterpretation of state sexual education policies, and removal of health education as a graduation requirement (24–26). Many of these barriers are present nationally as well (27–29).

Many dissemination models for EBPs have been developed for various health topics, and some have been applied for sexual health (30–40). These models have some limitations, however, and have not been particularly successful in helping school districts disseminate sexual health EBPs. First, existing models provide guidance for adopting and implementing EBPs in *community* settings rather than *school* settings. Consequently, these models may be less helpful to school districts that are often characterized by complex organizational structures and decision-making processes (41–43). This complexity can make it challenging, in particular, for program administrators, teachers, and other stakeholders to use EBPs in schools. Examples of such tasks include getting district-level approval to adopt and implement the program, competing against other district priorities (e.g., standardized testing), and coordinating implementation across several campuses that have a diverse set of resources. Second, most models (e.g., ADAPT-ITT, McKleroy et al.’s framework) (35, 36) stress *adaptation* of EBPs to fit the target population’s needs and culture, which may not be practical for school districts, versus *replication* of EBPs. Program adaptation requires knowledge of how to balance intervention fit with program fidelity (44), as well as sufficient time and resources to pilot test the adaptation (35, 36, 44), which many school districts often lack. In addition, because of the sensitive nature of the topic, an adaptation to a sexual health EBP that is improperly conducted could potentially negatively impact students. Subsequently, a program that is not properly adapted may not produce the same results as the original EBP (44). Thus, a more practical option for school districts may be to replicate an EBP with fidelity. Third, previous models (e.g., RE-AIM, Getting to Outcomes) (30, 38) provide theoretical guidance on *what* needs to be accomplished to adopt, implement, and maintain EBPs (or change some general behavior, as in the transtheoretical model) (39), but they do not give step-by-step direction on *how* to complete these tasks, particularly in school settings. This type of practical guidance is critical for the successful dissemination of sexual health EBPs in complex school settings.

**Figure 1:**
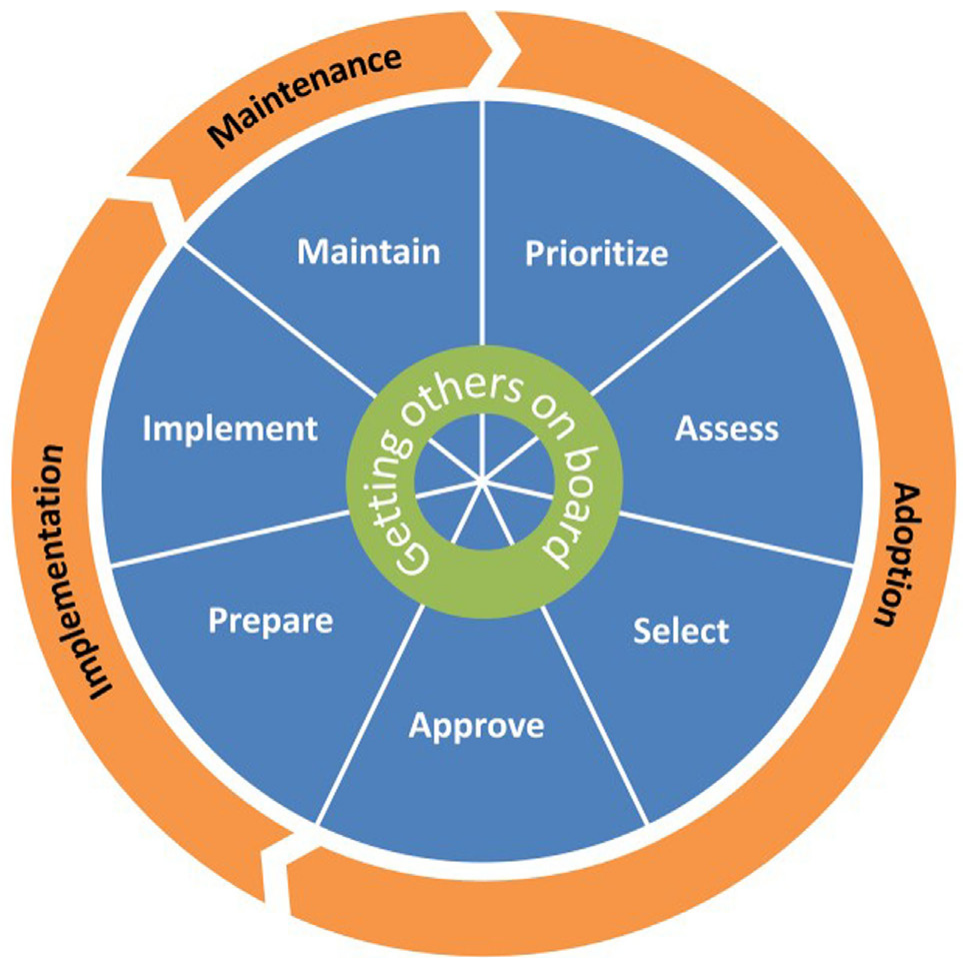
The *CH*oosing *A*nd *M*aintaining Effective *P*rograms for *S*ex Education in *S*chools Model.

**Figure 2:**
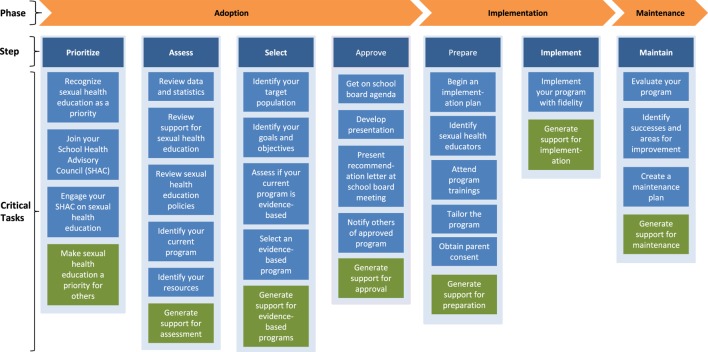
Steps and critical tasks for each phase in the *CH*oosing *A*nd *M*aintaining Effective *P*rograms for *S*ex Education in *S*chools Model.

To overcome limitations of these previous dissemination models, we used intervention mapping (IM), a six-step systematic process that uses theory, empirical evidence, and community input (44) to develop the *CH*oosing *A*nd *M*aintaining Effective *P*rograms for *S*ex Education in *S*chools (*CHAMPSS*) Model. Specifically, we used IM Step 1 (*conduct logic model of the problem, including needs assessment*) and Step 2 (*develop matrices of change objectives for each behavioral outcome*) to develop the model. The development of the model using these IM steps has been described in detail elsewhere (25). Briefly, we developed three matrices for three behavioral outcomes—adopt, implement, and maintain—which ultimately informed the development of the *CHAMPSS* Model described below and in Figures 1 and 2. As an example, a partial IM matrix for the adopt behavioral outcome is provided in Table 1.

**Table 1:** Partial intervention mapping Step 2 matrix of change objectives for adopt behavioral outcome^a^: school district board members will adopt (i.e., vote to approve) a sexual health evidence-based program (EBP).

	Determinants^c^^,^^d^				
Performance objectives (PO)^b^	Awareness/knowledge (A/K)	Attitudes (A)	Skills and self-efficacy (SSE)	Outcome expectations (OE)	Perceived norms (PN)
PO.1. Sexual health advocate will attend a School Health Advisory Committee (SHAC) meeting when discussions of adolescent sexual health education are taking place	A/K.1.a. Identify chair of SHAC to obtain SHAC meeting schedule	A.1.a Describe effort to attend SHAC meetings and to collaborate with the SHAC as essential to adopting an EBP		OE.1.a. State that attending SHAC meetings when sexual health education is discussed will lead to increased knowledge of current sexual health education practices and opportunity to advocate for EBPs in his/her district. OE.1.b. State that attending SHAC meetings when sexual health education is discussed will lead to increased support for EBPs in his/her district	PN.1.a. Recognize that other sexual health advocates attend SHAC meetings in decision making of sexual health curricula

PO.4. Sexual health advocate will engage his/her SHAC on sexual health education by including sexual health education on a SHAC meeting agenda	A/K.4.a. Describe the steps needed to include an item on a SHAC meeting agenda	A.4.a. Feel positive about including sexual health education on the SHAC meeting agenda	SSE.4.a. Feel confident in ability to include sexual health education on the SHAC meeting agenda	OE.4.a. Believe that including sexual health education on the SHAC meeting agenda will result in greater SHAC engagement on sexual health education	PN.4.a. Believe other sexual health advocates engage their SHACs on sexual health education by including sexual health education on a SHAC agenda

PO.5. SHAC will review current data and statistics on teen pregnancy and HIV/sexually transmitted infection (STI) in its district/school	A/K.5.a. List resources where current data and statistics can be obtained, and do so A/K.5.b. Summarize sexual behavior, HIV, STI, and pregnancy statistics among students	A.5.a. Describe review time as necessary and important A.5.b. Believe sexual health education is a priority for his/her school district	SSE.5.a. Demonstrate ability to find statistics related to sexual health in his/her city/district SSE.5.b. Correctly interpret statistics related to sexual health SSE.5.c. Feel confident in interpreting sexual behavior, HIV, STI, and pregnancy statistics	OE.5.a. Expect that evaluating student statistics of HIV, STI, and pregnancy will result in a better understanding of the needs and priorities regarding sexual health education in the district	PN.5.a. Recognize that other districts and their own students, families, teachers, and principals see teen pregnancy, HIV, and STIs as a problem that needs to be addressed PN.5.b. Perceives that other districts are reviewing most current data to identify the need for effective sexual health education

PO7. SHAC will identify the goals, target population, and desired outcomes regarding middle and high school (adolescent) sexual health education	A/K.3.a. List the health learning objectives (TEKS) for middle and high school students by grade level A/K.3.b. Describe desired students (age, ethnicity, and gender) to participate in a curriculum A/K.3.c. List desired goals (e.g., reduce teen pregnancy/HIV/STIs, decrease dropout rates, increase academic performance)	A.7.a. Describe identifying goals and target population as necessary and important to student health	SSE.3.a. Demonstrate ability to create effective goals for sexual health education SSE.3.b. Feel confident in identifying goals, target population, and outcomes for sexual health education in his/her district	OE.3.a. Expect that identifying goals and target population will help lead to a reduction in adolescent and teen HIV/STI/pregnancy, decrease dropout rates, and increase academic performance among students OE.3.b. Describe how identifying goals, target population, and desired outcomes will help identify the most appropriate EBP that will fit the needs of the district	PN.7.a. Recognize that school board, superintendents, principals, teachers, and parents share these goals and desired outcomes

PO.8. SHAC will review current state/district/school policy regarding adolescent sexual health education	A/K.8.a. Obtain and describe state policy on middle school sexual health education A/K.8.b. Obtain and describe district policy on middle school sexual health education A/K.8.c. Obtain and describe individual school policies on middle school sexual health education	A.8.a. Feels positive about reviewing state/district/school policy regarding sexual health education	SSE.8.a. Summarize the state/district/school policy for sexual health education and implications for implementing an EBP SSE.8.b. Feel confident in interpreting state/district/school policy regarding sexual health education	OE.8.a. Describe how reviewing state/district/school policies will result in adopting an EBP that is in accordance with district policy	PN.8.a. recognizes that other district ally and sexual health advocates are reviewing current policy and making sure it reflects what is necessary given current statistics

PO.11. SHAC will determine if the district is currently implementing a pregnancy/HIV/STI curriculum(s) and if so, will review and assess whether the curriculum(s) is evidence-based and meets the identified goals and objectives	A/K.11.a. Describe what an EBP is (has program evaluations that are experimental in nature, participants are randomly assigned to treatment and control groups, focus on changes in the behavior of program participants, etc.) A/K.11.b. List advantages of EBPs and why they are important to implement	A.11.a. Feel positive about EBPs	SSE.11.a. Demonstrate ability to identify EBPs when presented with a non-EBP SSE.11.b. Feel confident in identifying EBPs	OE.11.a. State that EBPS will lead to desired behavioral change among students	PN.11.a. Believe that other districts are changing to EBPs, which are important for student behavior change

PO.12. SHAC will review and evaluate evidence-based pregnancy and HIV/STI prevention program(s) available to the school district that meet the goals, target population, and desired outcomes	PO.12.a. SHAC will find EBPs	A/K.12a.a. Describe where to find EBPs	A.12a.a. Feel positive about finding EBPs	SSE.12a.a. List EBPs SSE.12a.b. Feel confident in finding EBPs		

PO.13. SHAC will elicit support of potential EBPs with other district sexual health advocates, principals, parents, and community members, discussing feasibility and resources required	A/K.13.a. List strategies for obtaining support for EBP adoption A/K.13.b. List barriers that may derail support A/K.13.c. List possible strategies for overcoming adoption barriers	A.13.a. Feel positive about eliciting support and overcoming barriers	SSE.13.a. Demonstrate ability to use strategies for increasing support of EBPs SSE.13.b. Feel confident in eliciting community support for EBPs	OE.13.a. State that endorsement of EBPs by key stakeholders will lead to increased probability of adoption of EBPs by district/school leaders	PN.13.a. Recognize that other SHACs have overcome these obstacles and successfully elicited support for such programs

PO.14. SHAC members will create and present a position statement/paper with recommendations for sexual health education in their district, including recommending school board approval of curriculum(s)	A/K.14.a. List components of an effective statement/position paper A/K.14.b. List recommended EBP(s) for school board approval A/K.14.c. List effective strategies for presenting the recommended EBP(s) to the school board (e.g., formal presentation at a board meeting)	A.14.a. Feel positive about recommending an EBP program to school board	SSE.14.a. Demonstrate ability to write components of a position statement SSE.14.b. Demonstrate ability to develop a presentation for school board SSE.14.c. Feels confident in recommending EBP to School board through position statement and presentation	OE.14.a. Describe how creating a position statement may lead to increased support for selected EBP by school board OE.14.b. Describe how giving a presentation to the school board regarding recommended EBP(s) will lead to an opportunity to advocate for the selected EBP(s)	PN.14.a. Believe other SHACs create position statements with recommendations to school board for EBPs

PO.16. School board members will adopt (an) evidence-based pregnancy and HIV/STI prevention program(s)	A/K.16.a. Describe process for school board approval A/K.16.b. List recommended EBPs approved	A.16.a. Feels positive about adopting an EBP			PN.16.a. Notes that other districts are adopting EBPs, which are important for student behavior change

*^a^This matrix informed the development of the CHAMPSS Model adoption phase in Figure 1*.

*^b^POs are the sub-steps required to complete the behavioral outcome; informed the development of the prioritize (PO 1, 4), assess (PO 5, 7, 8), select (PO 7, 11, 12, 13), and approve (PO 4, 16) steps and associated critical tasks within the *CHAMPSS* Model adoption phase in Figure 2*.

*^c^Determinants are identified from theory and empirical evidence for the behavioral outcome and POs*.

*^d^Change objectives are written where rows and columns intersect and ask the question “What needs to change in determinant ‘X’ for an individual to do PO ‘Y’ (44).” There may be more than one change objective per cell (designated as a, b, etc.)*.

The *CHAMPSS* Model comprises three phases: “adoption,” “implementation,” and “maintenance” and seven steps: “prioritize,” “assess,” “select,” “approve,” “prepare,” “implement,” and “maintain.” A core element is “Getting Others on Board” (i.e., connecting with other supporters of EBPs and adolescent sexual health), which extends across all seven steps (Figure 1). Akin to some previous models (e.g., RE-AIM, Getting to Outcomes) (30, 38), the *CHAMPSS* Model is circular because school district stakeholders may enter the model at any step, depending on their readiness. Furthermore, school district stakeholders may complete one step but then realize that they need to go back to a previous step. A unique feature of the model, though, is the corresponding list of prescribed critical tasks that must be accomplished to complete a step (Figure 2). As an example, the performance objectives (PO) (sub-steps required to complete each behavioral outcome) (44) in Table 1 informed the development of the prioritize, assess, select, and approve steps and associated critical tasks within the *CHAMPSS* Model adoption phase in Figure 2.

Key stakeholders, who have the authority and ability to adopt, implement, and maintain sexual health EBPs in the school setting, are included throughout the CHAMPSS Model (25). Key adopter stakeholders may include members of the school district’s Board of Trustees (who vote to approve adoption of a sexual health EBP in the school district) and School Health Advisory Committee (SHAC, a school district subcommittee which includes parents—required by law in Texas—and makes health-related program recommendations to the school board) (45). Key implementers may include individuals at the school district level (e.g., a district curriculum coordinator) and at the local school level (e.g., principals, school curriculum coordinators, and teachers). Individuals responsible for maintaining implementation of a sexual health EBP may include district and school curriculum coordinators. In addition, any other person who is interested in and committed to supporting the dissemination of a sexual health EBP (a “sexual health advocate”) can be an adopter, implementer, or maintainer.

The CHAMPSS Model overcomes limitations of previous dissemination models because it specifically targets school district stakeholders; stresses replication with fidelity; and provides detailed, step-by-step instructions that include realistic tasks for school district stakeholders to accomplish in each step of the model (25). Although the CHAMPSS Model provides a useful guiding framework for school district stakeholders, we envisioned the development of an online decision support system that further operationalizes the steps of the model. This online decision support system would specifically guide users through the prescribed critical tasks within the CHAMPSS Model, provide resources and skills specific to each task, provide technical assistance to help overcome barriers, and foster linkages with users in other school districts. Thus, the purpose of this “Methods” paper is to describe how we used IM Steps 3, *Program Design*, and 4, *Program Production*, to develop this online decision support system, ultimately named *iCHAMPSS* (so named because it was the *interactive* version of the CHAMPSS Model).

## Methods

The *iCHAMPSS* development group was an academic-community-health department collaboration that was formed during the development of the *CHAMPSS* Model (25). Group members comprised study investigators (epidemiologists, behavioral scientists, psychologists); masters-level staff trained in public health; county health department representatives; and the “*CHAMPSS* Group,” a group of school-based community stakeholders who themselves adopted the name of the model. Briefly, the CHAMPSS Group comprised individuals from a subgroup of the Harris County School Health Leadership Group that was convened by the Harris County Public Health department. This subgroup included 22 members and was formed to specifically help school districts in Harris County adopt and implement sexual health EBPs. Members of the CHAMPSS Group represented 15 area school districts and included parents, nurses, science curriculum coordinators, and SHAC representatives. The CHAMPSS Group met together bimonthly; study investigators and/or staff often presented at their meetings to provide skills related to using and implementing sexual health EBPs (e.g., developing program objectives, finding data on adolescent sexual health, and assessing parental support for EBPs) as well as to garner buy-in and input for *iCHAMPSS* intervention development. By working with the CHAMPSS Group, we hoped to ensure the development of a user-friendly and useful online decision support system for school districts.

To develop *iCHAMPSS*, the development group completed Steps 3 (*Program Design*) and 4 (*Program Production*) of IM (44). In Step 3, we used theory and empirical evidence to (a) identify intervention delivery vehicles and program themes; (b) identify theoretical methods and practical applications for each group of change objectives, organized by determinants, for each behavioral outcome; and (c) draft a program scope and sequence. According to Bartholomew and colleagues, “a theory- and evidence-based change method is a general technique for influencing the determinants of behaviors….” Practical applications include the intervention strategies used to operationalize those methods. It was also important to specify the “parameters” or situations under which a method is used appropriately. In Step 4, we used our methods and applications from Step 3 to (a) refine the *iCHAMPSS* program structure and organization; (b) prepare plans for program materials; (c) draft messages, materials, and protocols; and (d) pretest, refine, and produce materials. This study was approved by the University of Texas Health Science Center at Houston Institutional Review Board.

## Results

### Step 3: Program Design

#### Program Delivery Vehicle and Theme

As part of the initial design process for *iCHAMPSS*, we first identified the delivery vehicle by which we would operationalize the CHAMPSS Model. Early on, during IM Steps 1 and 2, we had decided that we would use the Internet to create an online decision support system to accomplish this task. The Internet is widely used to transmit information, and members of the CHAMPSS Group agreed that a website would be the most efficient way to provide access to the *iCHAMPSS* tools and resources. Furthermore, the Internet has been widely used to disseminate information about sexual health EBPs (13, 14), although this information predominantly focuses on describing EBPs, providing program materials, and linking users to training resources. In addition, other online decision support systems have been designed to help health care providers make decisions regarding their patients’ symptoms and treatment plans within the clinical arena (46, 47). Recently, for example, an online decision support system was developed in Canada to promote the use of research evidence to inform decisions regarding public health (48). We also identified a program theme for the online decision support system, which was to be a sexual health advocate, or champion, for the dissemination of sexual health EBPs by school districts. Finally, we created an *iCHAMPSS* logo, which incorporated the round CHAMPSS Model, and the byline of the *iCHAMPSS* website (which appears on every web page) is “*CH*oosing *A*nd *M*aintaining Effective *P*rograms for *S*ex Education in *S*chools.”

#### Methods and Practical Applications

To begin the process of developing specific activities for *iCHAMPSS*, we identified methods, parameters, and practical applications. We used several behavioral science theories, including theories of information processing (44, 49), social cognitive theory (44, 50, 51), and diffusion of innovations (17) to identify specific methods for each group of change objectives, organized by determinants, for each behavioral outcome. Table 2 provides examples of methods, parameters, and practical applications for each critical task from the CHAMPSS Model adoption phase-select step (informed by the adopt behavioral outcome matrix provided in Table 1). For example, in task 1 (identify the target population), theories of information processing (44, 49) informed our use of elaboration as a theoretical method to influence change objectives associated with increasing awareness and knowledge related to describing the target population (age, ethnicity, and gender) for the EBP curriculum (change objectives A/K.7.b). In addition, modeling from social cognitive theory (44, 50, 51) was used to help promote more favorable attitudes and outcome expectations (OE) related to identifying the target population (change objectives A.7.a, OE.7.a, OE.7.b).

**Table 2:** Partial intervention mapping Steps 3 and 4: identifying methods, parameters, practical applications, tool types, and example messages for each critical task from the CHAMPSS Model adoption phase-select step.

CHAMPSS Model critical tasks^a^	Determinants and change objectives^b^	Methods^c^	Parameters^c^	*iCHAMPSS* practical application^c^	*iCHAMPSS* tool type	Example messages in *iCHAMPSS*
1. Identify your target population	Awareness/knowledge (A/K.3.b) Attitudes and outcome expectations (OE) (A.7.a, OE.3.a, OE.3.b)	Elaboration Persuasive communication Modeling	Messages must be personal, understandable, and highly relevant for users, individuals must be motivated to receive messages Messages must be relevant, not too dissimilar from user, often repetitive Model must be relatable, describe specific steps or skills, and receive reinforcement	– Video/animated tutorial by an expert on selecting EBPs that covers identifying target population for desired outcomes – Recommendation letter to school board which includes identified target population – Video testimonials of School Health Advisory Committee (SHAC) members and other school personnel discussing how/why they selected the target population for their district	Step overview Templates Success stories	“*Step overview*”: “…what population do you want to serve? Select a program that was tested among a similar population to that in your district. It will be more likely to have a similar impact on *your* students. Determine if there’s a certain school or a particular grade level in which teen pregnancy is most prevalent” “*Success stories*”: “… our initial goal was to have a program from sixth through ninth grade… we figured if we can get them in seventh and eighth grade, we can kind of nip some of the risky behaviors in the bud before they have that transition over the summer as eighth graders going to ninth grade and then starting their freshman year in high school”

2. Identify your goals and objectives	Awareness/knowledge (A/K.3.a, A/K.3.c) Skills and self-efficacy (SSE) (SSE.3.a, SSe.3.b) Attitudes OE Perceived norms (PN) (A.7.a, OE.3.a, OE.3.b, PN.7.a)	Elaboration Goal-setting Persuasive communication Modeling	Messages must be personal, understandable, and highly relevant for users, individuals must be motivated to receive messages Being committed to achieving goals Messages must be relevant, not too dissimilar from user, often repetitive Model must be relatable, describe specific steps or skills, and receive reinforcement	– Video/animated tutorial by an expert on selecting EBPs that covers setting goals – Recommendation letter to school board which includes identified goals – Internet links to interactive exercises on identifying and creating goals and objectives – Video testimonials of school personnel discussing the goals and objectives their district identified	Step overview Templates Helpful links Success Stories	*Helpful links*: Centers for Disease Control and Prevention—Communities of Practice—SMART Objectives Template Download this “SMART Objectives Template” from the Centers of Disease Control to quickly develop your own SMART (specific, measurable, achievable, realistic, time-bound) objectives (http://www.cdc.gov/phcommunities/resourcekit/evaluate/smart_objectives.html)

3. Assess if your current program is evidence based	Awareness/knowledge (A/K.11.a, A/K.11.b)	Elaboration	Messages must be personal, understandable, and highly relevant for users, individuals must be motivated to receive messages	– Video/animated tutorial by an expert on selecting EBPs that covers how to assess whether a district’s current program is evidence based – Internet links to national lists of sexual health EBPs	Step overview Helpful links	“*Step overview*”: “Are any sexual health education programs currently used in your district on the list? A program may be *labeled* as evidence-based or a vendor may have *told* you the program has evidence but if it’s not on the list it’s probably *not* evidence based”

4. Select an evidence-based program (EBP)	Awareness/knowledge (A/K.12a.a) SSE (SSE.11.a, SSE.11.b, SSE.12a.a, SSE.12a.b) Attitudes OE PN (A.11.a, OE.11.a, PN.11.a, A.12a.a)	Elaboration Technical assistance Persuasive communication Modeling	Messages must be personal, understandable, and highly relevant for users, individuals must be motivated to receive messages Must fit the user’s needs Messages must be relevant, not too dissimilar from user, often repetitive Model must be relatable, describe specific steps or skills, and receive reinforcement	– Video/animated tutorial by an expert on selecting EBPs that covers where to find EBPs – Fact sheet on characteristics of EBPs – Fact sheet on how to interact with non-EBP vendors (being a smart shopper) – Internet links to national lists of EBPs that provide guidance on how to select the best EBP for one’s setting – Video testimonials of SHAC members and other district why EBPs are beneficial and important	Step overview Facts and tips Facts and tips Helpful Links Success stories	From *“Smart Program Shopping” facts and tips* *Select a sexual health education program if it*… – Is listed on a respected registry for EBPs (e.g., Office of Adolescent Health, National Campaign to Prevent Teen and Unplanned Pregnancy) – Was tested among a population with similar demographics to those of your district or school (e.g., gender, race/ethnicity, grade) – Was effective in changing the behaviors you want to target (e.g., delay sexual initiation, increase condom or contraceptive use) – Meets your district or school’s goals and objectives – Reflects values consistent with those in your district or school

5. Generate support for EBPs	Attitudes OE PN (A.13.a, OE.13.a, PN.13.a) SSE (SSE.13.a, SSE.13.b)	Persuasive communication Modeling Shifting perspective Technical assistance	Messages must be relevant, not too dissimilar from user, often repetitive Model must be relatable, describe specific steps or skills, and receive reinforcement Model must be able to take the perspective of the learner Must fit the user’s needs	– Video testimonials of SHAC members and other district parents/personnel describing how they get support for EBPs – Skills and tips on communicating effectively with others – Practice worksheet for analyzing key audiences for messaging on EBPs	Success stories Facts and tips Templates	“*Success Stories*”: “…And what I’ve learned is that if you address these issues that are potentially controversial in a very straightforward manner you have information readily available for people who have questions. You’re able to rebut any myths that come up quickly. And to have an external resource that can help you as well in answering those questions—an expert, someone who can also give data about the reality for kids who don’t get this information and just being pretty frank and up front and not stopping your march forward just because you’re afraid that something negative is going to occur”

*^a^From Figure 2*.

*^b^Determinants and changes objectives from the adopt behavioral outcome matrix in Table 1*.

*^c^A theory-and evidence-based method “is a general technique for influencing the determinants of behaviors…”; parameters refer to the situations under which a method is used appropriately; practical applications include the intervention strategies used to operationalize those methods (44)*.

Next, we brainstormed practical applications that would correspond to each theoretical method. For elaboration (theoretical method), we included a video/animated tutorial by an expert on selecting EBPs that covered identifying the target population for desired outcomes (practical application). Experts were members of the *iCHAMPSS* development group who were personal, motivating, understandable, and relevant to each school district, which were parameters for elaboration. To change attitudes and OE using modeling (theoretical method), we recommended a video-based testimonial of SHAC members and other school personnel discussing how and why they selected the target population for their school district (practical application). Models were similar to potential *iCHAMPSS* users (and, thus, relatable), explained the specific steps they used to overcome challenges, and expressed receiving reinforcement for their decisions (44), which were all parameters for modeling. Table 2 (first five columns) provides additional examples of the methods, parameters, and practical applications for the five critical tasks from the CHAMPSS Model adoption phase-select step (Figure 2), organized by determinants and change objectives from Table 1.

We also solicited input on other practical applications from the CHAMPSS Group. CHAMPSS Group members, in particular, requested a discussion forum to learn about what other school districts were doing. This suggestion corresponds to the method of interpersonal networking, which has been shown to facilitate adoption of effective programs by later users (17, 52, 53). CHAMPSS Group members also requested customizable templates that they could use, such as needs assessment and program evaluation forms, as well as tips sheets on engaging administrators, school board members, and others in getting district support for sexual health EBPs. These practical applications correspond to the method of facilitation from social cognitive theory (44, 50). The “Stage Your District” tool was another practical application suggested by the CHAMPSS Group. The original concept for this tool came from our partner at Harris County Public Health who had developed a simple four-question worksheet that would allow districts to determine in which stage of the CHAMPSS Model they were, indicating their level of readiness to adopt, implement, or maintain sexual health EBPs. At the beginning of every CHAMPSS Group meeting, members would stage themselves informally in the CHAMPSS Model, which they found useful for staying on track and monitoring progress. The “Stage Your District” practical application corresponds to the method of tailoring from the transtheoretical model (39, 44).

After examining the list of practical applications for all CHAMPSS Model critical tasks, organized by determinants and change objectives from each matrix, we identified some commonalities in the types of applications being proposed. These common practical applications included step overviews, success stories (videos/testimonials), facts and tips, helpful Internet links to outside resources, and templates. Next, we organized the list of practical applications by specific topical areas (e.g., sexual health priority, needs assessment, state law, and SHACs) to narrow the list and determine if there was any overlap among applications. These practical applications became known as the five types of “tools” available in *iCHAMPSS*. Table 2 (sixth column) provides an example of how the tool types were matched to practical applications.

#### Scope and Sequence

We described the scope and sequence of *iCHAMPSS* as self-directed and self-paced. However, we recommended that users (e.g., school district stakeholders) first complete the staging tool, “Stage Your District,” which would direct them to specific tools based on their level of readiness to adopt, implement, or maintain sexual health EBPs. Message tailoring is recognized as a crucial element in the creation of effective interactive health promotion programs (54). *iCHAMPSS* was also designed to allow users from any step of the CHAMPSS Model the flexibility to use whichever *iCHAMPSS* tools they deem most appropriate at any given time.

### Step 4: Program Production

#### Refining Program Structure and Organization

**Figure 3:**
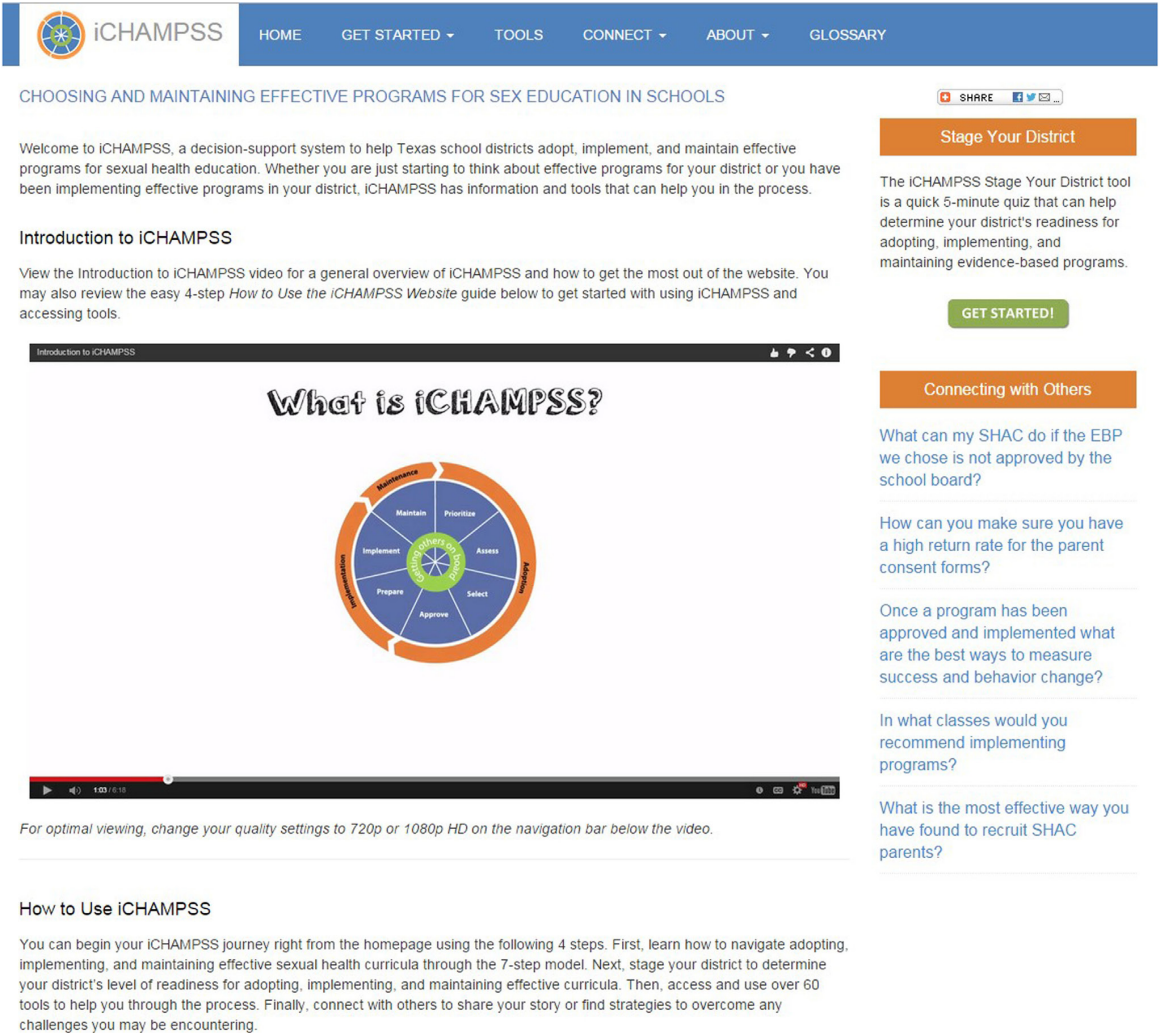
Screenshot of the iCHAMPSS introductory video tutorial.

The goal of *iCHAMPSS* was to provide decision support to aid sexual health advocates in adopting, implementing, and maintaining a sexual health EBP in their district/school. To that end, *iCHAMPSS* included *four features*. The first feature, the CHAMPSS Model description, was designed to familiarize users to the CHAMPSS Model. In addition to introducing users to this conceptual framework, there is a video-based tutorial that guides users through the *iCHAMPSS* process to learn how all the *iCHAMPSS* tools and CHAMPSS Model steps fit together (see Figure 3). The second feature comprises the staging tool described in IM Step 3. Also described in IM Step 3, the third feature, the tools library, disseminates useful information and forms through the five iCHAMPSS “tool types” (step overviews, success stories, fact and tips, helpful Internet links, and templates). Users do not need to understand the CHAMPSS Model to find and use the tools. Finally, the fourth feature (also described in IM Step 3) comprises a linkage system, or online discussion board, to help users communicate with other users across school districts and receive technical assistance from experts in the field.

#### Preparing Plans for Program Materials

As part of IM Step 4, we developed design documents for *iCHAMPSS’s* features, website, and tools. Design documents are detailed planning documents that instruct the program designers on how to produce the program materials (44). For *iCHAMPSS’s* features and website, we developed a 35-page design document outlining their specifications. In this design document, we specified several critical features of *iCHAMPSS* (available online at http://www.iCHAMPSS.org), including how it would be accessible to school district stakeholders, data collection features (only basic site usage data to be collected but user sign-in is necessary for the discussion board), hosting and maintenance capabilities, development constraints (e.g., server specificities and compatibility with all Internet browsers), and user interface frame (i.e., header, dashboard, content space, and footer). We also specified what users would see on the *iCHAMPSS* homepage: the name and an introduction to the system, photographs indicating that the focus of the site is on the health of adolescents, social networking share buttons, and direct links with short descriptions to the CHAMPSS process, the tools library, and the connect with others forum. Embedded within the CHAMPSS process page is an image of the circular, seven-step CHAMPSS Model, which expands to give a description of each phase or step when the user clicks on that part of the model.

For the five tool types, we also developed design documents that specified the purpose/main objectives of the tools, target audience/likely users, instructions for use, format (e.g., PDF file and video), and detailed description of tool content. For each tool type, the goal was to develop a variety of tools mapped to each CHAMPSS step and associated critical tasks.

#### Drafting Messages, Materials, and Protocols

**Figure 4:**
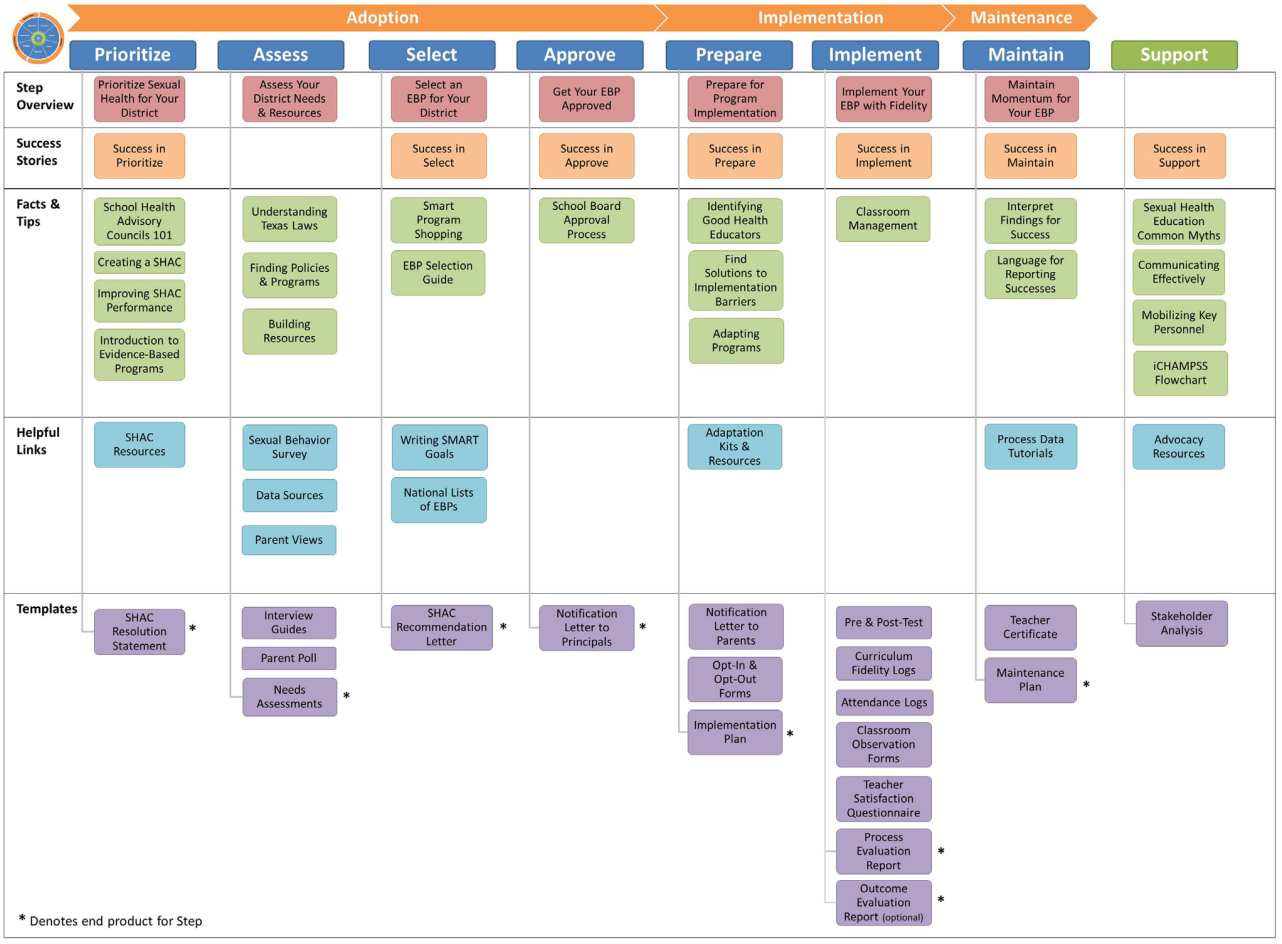
iCHAMPSS tools library.

**Figure 5:**
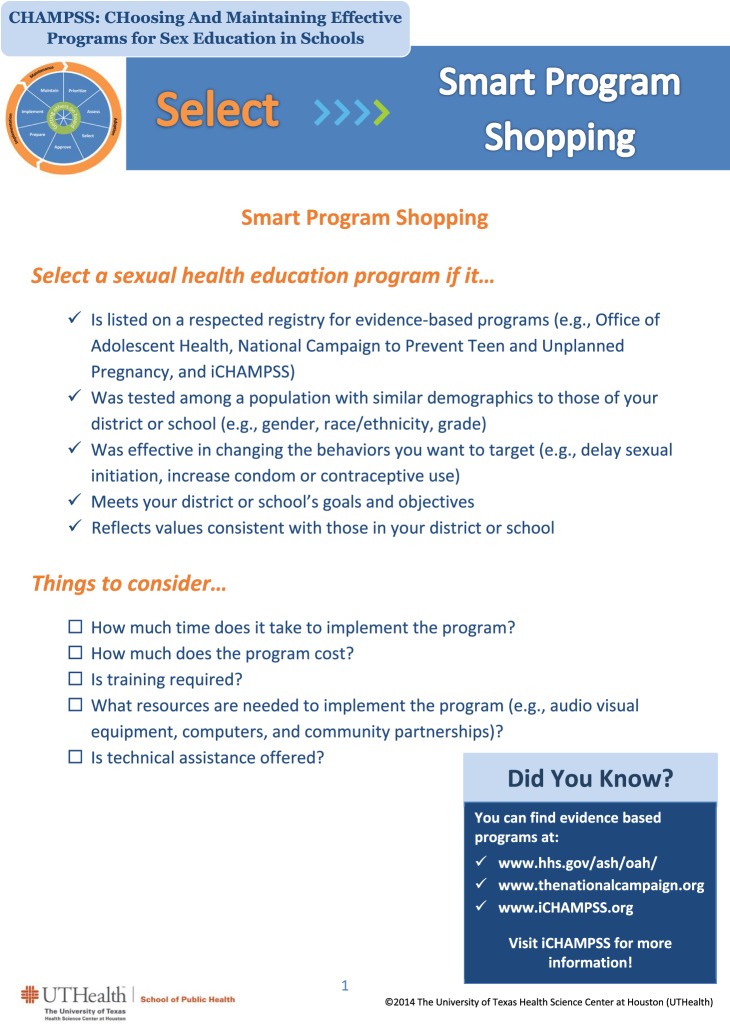
Screenshot of iCHAMPSS facts and tips tool type.

We procured the services of an IT developer, computer graphic designer, video production, and post-production specialists to design and produce *iCHAMPSS’s* website, features, and tools. However, we produced the content for the tools in-house, including the “Stage Your District” tool. We wrote the messages within each tool with the goals of accomplishing each critical task outlined in the CHAMPSS Model; these critical tasks were previously identified through the PO in each of the intervention matrices. One or two project team members initially developed each tool’s content, but we regularly included discussion of the tools to assess progress and address development questions and concerns in weekly meetings. Table 2 (seventh column) provides an example of how methods, parameters, and practical applications were operationalized to produce example messages that were incorporated into each of the tool types. During the development of messages, we also specified one of the templates as the “end product” for each CHAMPSS step that signified that step’s completion. We produced 62 tools, which are listed in a “tools library” on the *iCHAMPSS* website (Figure 4). See Figure 5 for screen capture of a selected facts and tips tool. Because we were producing a variety of tools with different delivery vehicles, the purpose and development approach varied for each tool (see Table 3 for a summary).

**Table 3:** *iCHAMPSS* “Stage Your District” tool and tool types^a^: delivery vehicles, purpose, description, and development.

Category	Delivery vehicle	Purpose	Description	Key development tasks
“Stage Your District”	Internet	To provide users with tailored feedback related to the step of the CHAMPSS Model their school district was currently in	21 questions mapped to the seven CHAMPSS Model steps User receives staging report that indicates where he/she should begin in *iCHAMPSS*	Develop staging algorithm (CHAMPSS Model matrices used to identify critical tasks from the model for successful completion of each step) Program staging algorithm Test tool to ensure algorithm and recommendations were logical based on the responses

Step overview	Video of expert lecturing with light whiteboard animation style	To provide users with an overview of each step and guide them through the basic knowledge and critical tasks required to complete each step of the CHAMPSS Model	7 step overviews—one for each step of the CHAMPSS Model Each approximately 5 min or less	Write script Identify visual text and graphics Film experts Edit video clips Create animation

Success stories	Video of evidence-based program end user	To provide users with video-based testimonials (role modeling stories and experiences) from school district stakeholders regarding challenges they faced or strategies used when going through a particular CHAMPSS step with their district	38 total from 17 school district stakeholders Each approximately 7 min or less Designed in an interview-style format in school-like settings Sample interview questions displayed at the beginning of each topic segment	Develop interview questions for stakeholders Identify interviewees from diverse school districts representing multiple district roles Film interviews and transcribe video Edit video content Finalize videos

Facts and tips	Print documents	To provide users with documents summarizing factual information or strategies critical to accomplishing the critical tasks in a particular CHAMPSS Model step	20 1–3 page, easy-to-read downloadable PDFs that could be printed	Write content Format document

Helpful links	Hyperlinks to external website	To provide users with relevant external website links relevant to accomplishing the critical tasks in a particular CHAMPSS Model steps	51 Internet links that provide helpful resources Included a thumbnail image of each website, a brief summary of resources on website, hyperlinks to website	Identify relevant websites

Templates	Print documents	To provide users with customizable documents that can be downloaded to aid in completion of CHAMPSS Model steps	19 Microsoft Word templates that varied in content and length; customizable so that they could be tailored to fit each district’s needs, data, and policies For each model step, one served as the “end product”	Write content Format document

*^a^Specific iCHAMPSS tools for each tool type are listed in Figure 4*.

#### Pretesting, Refining, and Producing Materials

Members of the CHAMPSS Group reviewed *iCHAMPSS* after it was initially completed. Overall, the majority felt the website was professional, informative, and visually pleasing. However, some felt that the image on the homepage was too scientific and that the homepage lacked sufficient information to draw visitors to the website. Their suggestions included the following: (a) placing a prominent description of *iCHAMPSS* on the homepage; (b) reorganizing the placement of some of the dashboard buttons, and (c) including upcoming national and local trainings and events. Members of the CHAMPSS Group also reviewed the “Stage Your District” tool and suggested changing the color scheme and enabling a print option. Some felt that the staging tool was unclear. Lastly, project team members reviewed *iCHAMPSS* and tested it in different Internet browsers and on mobile phones to ensure that it would function on different platforms.

**Figure 6:**
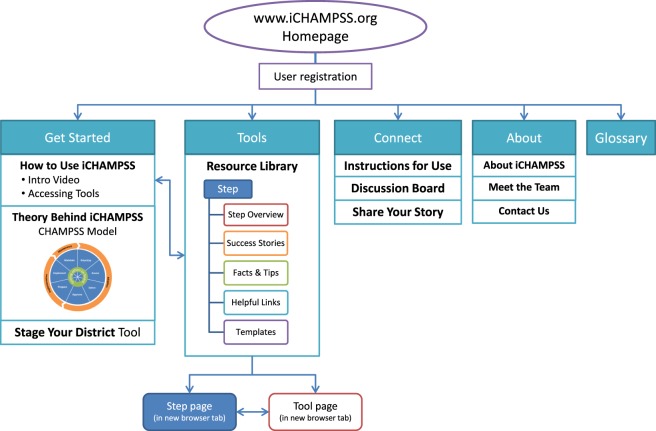
Final mock-up of iCHAMPSS online decision support system.

Based on these reviews, we made several changes to the website to clarify its purpose and specify where a user should begin. First, we revised the website name. Originally, CHAMPSS stood for “*CH*oosing *A*nd *M*aintaining *P*rograms for *S*ex Education in *S*chools.” However, it was important to add the word “Effective” before the word “Programs” because this was a distinguishing feature of the website. We added this new tagline to every page of the website, so that the purpose of the website was clear. Second, we modified the dashboard to clearly direct users to a “Get Started” button. The “Get Started” button included three drop-down menus: “How to use *iCHAMPSS*,” “Theory behind *iCHAMPSS*,” and “Stage Your District.” Third, we added a direct link to the “Stage Your District” tool on the homepage and modified its color scheme to be more clear and visually appealing. We also included a brief description of this staging activity so that users would understand its purpose. Stylistic modifications were also made to the entire website, including changing coloring to be consistent throughout the website and fixing spacing issues. A final mock-up of the *iCHAMPSS* online decision support system is provided in Figure 6.

Members of the CHAMPSS Group also provided feedback during the development process of various tools, mainly the end product templates and facts and tips. Most CHAMPSS Group members reported that the tools were useful, feasible, and likely to be used by their districts. After the *iCHAMPSS* tools were developed, three project team members successively reviewed each tool to assess its content, readability, and ability to meet intended objectives. The original tool developer(s) then finalized the tool content based on the feedback from all reviewers and submitted the tool for one final review and approval by the project team.

To further assess the usability, acceptability, and potential impact of *iCHAMPPS*, we also conducted a pilot study with 16 participants who were given access to *iCHAMPSS* for 3 weeks (55). During this time period, participants could download and use any of the tools on the website. They were asked to complete a web-based pretest and immediate posttest using Qualtrics survey software. Participants included professional staff from school districts and community organizations throughout Texas, and parents of school-aged children. To recruit participants, we distributed (*via* e-mail) a flyer describing the study to our community partners, which include the state health department, school districts, community organizations, the Texas School Health Advisory Committee, and The Texas Campaign to Prevent Teen Pregnancy. A description of the study was also placed within the “Friday Beat,” the state health department’s weekly newsletter. Participants received a $50 gift card for completing both surveys. In summary, 16 participants reported that *iCHAMPSS* was motivational, easy to use, trustworthy, and helpful. They also reported that their self-efficacy for obtaining approval to implement an evidence-based sexual health education program from the School District Board significantly increased as a result of using *iCHAMPSS*. Elaborate results of this pilot test are published elsewhere (55).

## Conclusion

Steps 3 and 4 of the IM process provided a systematic framework that was critical for translating the CHAMPSS Model (developed using IM Steps 1 and 2) into the *iCHAMPSS* online decision support system to help school district stakeholders adopt, implement, and maintain sexual health EBPs. Specifically, these steps provided a detailed process for ensuring that appropriate theoretical methods were identified and practical applications were developed to best meet the change objectives in the CHAMPSS Model matrices. The use of parameters, in particular, was most helpful in ensuring that the applications we chose best operationalized our chosen theoretical methods (44).

Some lessons can be learned from our experience developing *iCHAMPSS*. First, while we worked closely with the CHAMPSS Group to ensure the development of a system that was compatible with school district needs, time constraints prevented us from obtaining their feedback on every aspect of *iCHAMPSS*. For example, they never reviewed the final iteration of the discussion board, which was particularly disappointing because this feature was specifically requested by them. In addition, we received less feedback from the CHAMPSS Group on the implementation and maintenance plans because most group members did not have experience with these tasks (most CHAMPSS Group members were in the earlier stages of the CHAMPSS Model). It will be critical to obtain additional input on these tools from *future* iCHAMPSS users. Second, we learned important lessons about developing technology-based applications: (a) we did not anticipate the lengthy amount of time that would be needed to identify an appropriate IT developer and (b) it is important to adequately vet the IT developer to ensure that he/she has the requisite qualifications and communication skills. Regarding the former, the IT developer “search” process took approximately 9 months, which significantly delayed production of *iCHAMPSS*. For the latter, we had to hire a new IT developer mid-way through the development process because the original developer was not adequately meeting the needs of the project (e.g., the original “tools library” had to be rebuilt because the original programmed version did not allow us to add, delete, or format existing tools).

Future studies should focus on a rigorous evaluation of *iCHAMPSS* (IM Steps 5 and 6) to assess its impact on adoption, implementation, and maintenance of sexual health EBPs in school settings. This study should also assess the impact of *iCHAMPSS* on school district personnel’s psychosocial factors related to adoption, implementation, and maintenance, such as knowledge, attitudes, self-efficacy, and perceived support for sexual health EBPs. If effective in improving these outcomes, *iCHAMPSS* could serve as a model implementation science practice for sexual health EBPs nationally. Furthermore, *iCHAMPSS* could be adapted to increase dissemination of school-based EBPs that address other adolescent health issues, such as substance use and violence prevention.

## Ethics Statement

This study was carried out in accordance with the recommendations of “University of Texas Health Science Center at Houston Institutional Review Board” with passive informed consent from all subjects when required, in accordance with the Declaration of Helsinki. This publication only described our intervention development process. Verbal permission from the community advisory board partners for their input was obtained during the development process. The papers by Hernandez et al. (25, 55) did require passive informed consent which was required and stated in those manuscripts.

## Author Contributions

All authors (MP, BH, EG, PC, DL, ER, KR-H, YR, KJ-B, SE, and RS) made substantial contributions to the conception and design of the work; or the acquisition, analysis, or interpretation of the data; drafted the work or critically revised it for important intellectual content; provided final approval of the manuscript; and agreed to be accountable to all aspects of the work.

## Conflict of Interest Statement

The authors declare that the research was conducted in the absence of any commercial or financial relationships that could be construed as a potential conflict of interest.
